# Knowledge based automated planning for non‐coplanar VMAT stereotactic radiosurgery in ocular malignancies

**DOI:** 10.1002/acm2.70530

**Published:** 2026-03-03

**Authors:** Chase Cochran, Shane McCarthy, William St. Clair, Damodar Pokhrel

**Affiliations:** ^1^ Medical Physics Graduate Program Department of Radiation Medicine University of Kentucky Lexington Kentucky USA; ^2^ Indiana University Health System Radiation Oncology Indianapolis Indianapolis Indiana USA

**Keywords:** HARP model, HyperArc SRS, KBP model, ocular tumors, RapidPlan

## Abstract

**Background and purpose:**

Stereotactic radiosurgery/radiotherapy (SRS/SRT) has emerged as a less invasive alternative to enucleation in the management of ocular malignancies. SRS/SRT planning is time‐consuming, complex, and the plan quality depends on the experience of the treatment planner. We demonstrate the use of RapidPlan modeling in conjunction with HyperArc for the treatment of ocular diseases to improve planning efficiency, consistency, and quality.

**Materials and methods:**

A HyperArc‐based RapidPlan (HARP) model was iteratively trained on 80 patient datasets that simulated ocular malignancies. Twenty additional patient datasets were reserved for model testing and comparison with manual plans. Each testing dataset was both manually planned and replanned with the final RapidPlan model. Target volumes were defined in the HyperArc module as the GTV and PTV. The PTV was generated by a 2 mm static expansion of the GTV. 25 Gy in 1 fraction was prescribed, and all plans were normalized such that PTVD_95%_ = 25 Gy. Treatment planning was done using Eclipse V16 with the Acuros XB dose engine on a TrueBeam LINAC with Millennium 120 MLCs (5 mm width). All plans underwent EPID‐based patient‐specific QA and an independent Monte Carlo (MC) second‐check.

**Results:**

Model‐based plans demonstrated similar degrees of conformality, gradient, and target coverage compared to manual planning. OAR sparing showed statistically significant improvements with model‐based plans. Optic nerve Dmax (D0.03 cc) decreased to 4.56 Gy (manual: 7.18 Gy), and lens Dmax decreased to 7.64 Gy (manual: 9.36 Gy). Lacrimal gland mean dose decreased from 11.61 to 6.47 Gy. Modulation factor, monitor units, and beam‐on times also all decreased. Patient‐specific QA results showed improvements compared to manually generated plans. MC second check results also improved, increasing from an average of 97.81%–98.74%. Plan optimization times decreased significantly from approximately 120 min for manual planning to 15 min on average for model‐based plans.

**Conclusion:**

RapidPlan‐generated ocular SRS plans showed either comparable performance or improvements in all plan metrics measured, compared to manual planning. Planning times were significantly reduced while maintaining or improving plan quality. Clinical implementation of this HARP model is ongoing at our clinic.

## 1 INTRODUCTION

Various treatment methods currently exist to treat ocular disease. Treatment options include plaque brachytherapy, typically using Iodine or Ruthenium,^1^, ^2^, ^3^, ^4^ LINAC‐based external beam photon therapy,^5^, ^6^, ^7^ frameless or frame‐based stereotactic radiosurgery or radiotherapy (SRS/SRT) modalities like robotic CyberKnife,^8^, ^9^, ^10^ Gamma Knife radiosurgery,^11^, ^12^, ^13^ proton beam therapy,^14^, ^15^, ^16^, ^17^ and enucleation. Each modality has advantages and limitations. For example, brachytherapy excels in extremely conformal dose delivery but is not suitable for large tumors (thickness > 10 mm), and for tumors located near the optic nerve, plaque placement can be challenging. LINAC, CyberKnife and Gamma Knife SRS/SRT offer less invasive, high‐precision dose distributions as well, but tend to expose more of the surrounding normal tissues depending on beam geometry, margin setup, and fractionation compared to brachytherapy. Proton beam therapy has also been utilized with favorable results, but invasiveness remains an issue with the implantation of tantalum clips for motion management. The limited number of proton centers globally poses an additional issue with access for these patients as well. Enucleation, involving the surgical removal of the eye, provides similar survival rates as globe‐sparing strategies and, as a result, especially for small/medium‐sized tumors, globe‐sparing strategies such as radiotherapy are seeing more use.^18^ There is a clear need to develop additional treatment methods for this cohort that can deliver precise dose distributions, be minimally invasive, and be accessible to many institutions.

Advancements such as the introduction of HyperArc stereotactic radiosurgery have been used to improve conventional photon‐based SRS/SRT methods by simplifying the planning process, treatment delivery, and clinical workflow. For intracranial lesions and recurrent head and neck SRT, many studies comparing HyperArc versus conventional VMAT planning methods show favorable results.^19^, ^20^, ^21^, ^22^, ^23^, ^24^ The HyperArc module aims to streamline SRS/SRT planning with automatic arc geometry, isocenter placement, and collimator angle optimization. Manual plan optimization, however, remains the standard clinical workflow, with HyperArc and manual planning tends to be time‐consuming and subject to inter‐planner variability.

RapidPlan is a knowledge‐based planning system that uses machine learning models that were trained on previously approved treatment plans to predict achievable dose volume objectives for new plans. These generated objectives are then used to guide plan optimization, reducing reliance on manual trial and error during planning. RapidPlan has been shown to improve plan consistency and efficiency across multiple anatomical sites and treatment techniques.^25^, ^26^, ^27^ The combination of HyperArc and RapidPlan has also been reported in the literature^28^, ^29^; however, to the best of our knowledge, no study exists reporting a RapidPlan model developed for treating ocular SRS cases utilizing the HyperArc automated geometry. The purpose of this study was to develop and validate a RapidPlan model for the treatment of ocular malignancies with the HyperArc treatment platform. An ocular‐specific RapidPlan model, in conjunction with HyperArc, will allow for almost complete automation of the treatment planning process. This serves to further improve planning quality, safety, and efficiency for ocular SRS treatments with the HyperArc platform.

## 2 MATERIALS AND METHODS

### 2.1 Dataset creation

The basis for both the training and testing datasets was 100 anonymized HyperArc CT scans (acquired with the Encompass fixation device) that were gathered from our institutional database under IRB approval (IRB#92667). The target definition for these datasets posed a significant challenge. Our institution currently uses COMS plaques for ocular malignancies, leaving no datasets with both HyperArc‐compatible CT scans, which are acquired with the encompass fixation system, and an ocular malignancy available for targeting. To address this, we utilized two different approaches for target generation, one for our training dataset and another for our testing dataset.

### 2.2 Training dataset creation

Eighty anonymized, HyperArc‐compatible CT datasets were assigned synthesized tumor contours. Imaging utilized standard SRS protocols (512 × 512 image size, 1 mm slice thickness). The anonymized CT datasets were taken from patients originally treated for various brain malignancies at our institution. Although done out of necessity for this study, the synthetic tumor definition also allows for greater control over the training dataset than if we used existing tumor definitions. Synthesized contours were generated by sampling from normal distributions of COMS MRI‐derived tumor metrics (thickness, diameter, volume, etc.). Positioning variables (e.g., tumor laterality: left/right; position: anterior/posterior/lateral/medial) were randomly assigned to improve robustness in the training dataset. Target contours were then manually reconstructed onto the anonymized scans to replicate realistic target definitions in a wide array of proximity to organs at risk (OARs). Reconstruction of synthetic tumor contours required manually drawing, slice by slice, GTV volumes to fit the sampled tumor metrics. Random variation in tumor size or proximity OARs was added to enhance model robustness against non‐standard tumors.

### 2.3 Testing dataset creation

For the testing dataset, hybrid datasets were created by combining 20 anonymized CT scans, similar to those used in the training dataset, with 20 anonymized MRI scans from past COMS patients. MRI scans used an MPRAGE imaging sequence with gadolinium contrast‐enhancement and 1 mm slice thickness. Tumor contours from the COMS MRIs were manually re‐drawn slice‐by‐slice onto the HyperArc CT datasets, yielding hybrid datasets with clinically realistic tumor geometries that would be fully compatible with the HyperArc treatment planning in Eclipse.

### 2.4 Treatment planning

For treatment planning, organs at risk (OARs) were delineated, including the eyes, lenses, optic nerves, optic chiasm, lacrimal glands, brain, brainstem, and skin. The skin contour was defined by generating a 5 mm thick rind from the body contour. Planning target volumes (PTV) were generated by a uniform 2 mm expansion of the gross tumor volume (GTV) to account for setup uncertainty and intra‐fractional motion, consistent with established ocular SRS protocols.^5^, ^6^, ^7^ In addition to OARs, various control structures were generated as well, such as an 8 mm ring expansion on the PTV to control dose fall‐off and an Eye‐PTV structure used to push dose away from the distal part of the eye relative to the tumor. All SRS plans were generated using the Eclipse treatment planning system (TPS) V16 (Varian Medical Systems, Palo Alto, CA), with the HyperArc module. Automated arc geometry, isocenter placement, and optimized collimator rotation were utilized, with no intervention from the user, in order to standardize the treatment planning process. Plans were prescribed 25 Gy to the PTV in 1 fraction; all plans were normalized such that PTVD_95%_ = 25 Gy. A maximum dose objective was placed on the GTV at 125% prescription to try and minimize global maximums to <125%. Optic pathway sparing was of notable concern, as such optic nerve maximums were kept <12 Gy.^30^ Planning with HyperArc allows use of either the normal tissue objective (NTO) or SRS NTO. The SRS NTO helps minimize dose bridging in cases where multiple targets exist. The SRS NTO was used for all cases in this study since it is our standard protocol when using HyperArc. Since all plans used in this study were single‐lesion plans, the SRS NTO will act similarly to the normal NTO used in non‐HyperArc plans. For optimization, convergence mode was disabled, no MU objectives were set, jaw tracking was enabled, and aperture shape controller setting was set to low. All plans were designed on a TrueBeam linear accelerator using a 6MV flattening filter‐free (FFF) beam and a standard 120‐leaf Millennium MLC (5 mm width). Final dose calculations used the Acuros XB (V16.1.2) dose algorithm with GPU acceleration enabled using a 1.25‐mm dose grid size, reporting dose to medium.

### 2.5 Model training and validation

Model performance is highly dependent on both the quantity and quality of the training dataset. Fogliata et al.^31^ demonstrated an iterative training approach for developing a RapidPlan model, where plans generated by earlier model versions were used as training data for successive models. The second RapidPlan model, trained by the previous version showed further improvements in generated plan quality. Therefore, an iterative training approach was used to develop the final RapidPlan model presented in this study. Since the training datasets were synthesized, the process began with manually planning all 80 ocular SRS cases from the initial training dataset, using the same methodology described in the manual planning section. Following this, all 80 plans were attempted to be extracted to a RapidPlan model. Five of the plans were unable to be extracted due to a known memory limitation in Eclipse and were excluded.^32^ The remaining 75 plans were used to train the first version of the model (V1.0). After ensuring the model's performance was comparable, if not better, to the manually planned plans, the V1.0 model was used to replan the 75 training plans. The replanned plans were then extracted into a second RapidPlan model (V2.0). This process was repeated once more, with V2.0 generating 75 additional plans for training which were then extracted into a third RapidPlan model (V3.0). The third V3.0 model was analyzed using model analytics. Two outlier plans and several outlying structures were removed to improve dataset quality, resulting in the final V3.1 model. Optimization objectives in the final model included manually defined lower objectives on the PTV and GTV at 100% of the target volume receiving 100% and 105% of prescription respectively. An upper objective at 125% to limit global maximum doses was applied to the GTV. An additional manually set upper limit was on the optic nerve at 0% of the volume being limited to 9 Gy to try and limit max dose to the optic nerve. Model generated line objectives were applied to the brain, brainstem, lacrimal glands, lenses, chiasm, optic nerves, skin, and control structures. The final model was then evaluated by replanning the testing datasets and comparing them to the original testing plans.

### 2.6 Data collection and plan evaluation

All final ocular SRS plans were subjected to evaluation using both quantitative and qualitative metrics. Qualitatively, plans were reviewed by an experienced radiation oncologist and medical physicists. Dose distributions and target coverage were reviewed to ensure clinical acceptability. Quantitatively, all plans were evaluated using a variety of indices, including the gradient index (GI), Homogeneity index (HI) and Paddick conformity index (PCI).^33^, ^34^ Manual plans were generated very early during a feasibility study, and formal planning times were unfortunately not recorded. The planner therefore provided a conservative estimate of 120 min for manual plan optimization, which was used for comparison with model‐based planning times recorded for each case. Each of the treatment plans was evaluated via patient‐specific quality assurance (PSQA) using EPID‐based portal dosimetry.^35^ All PD plans were calculated using the PDIP V16.1.2 algorithm in Eclipse. The measured dose distributions from the EPID‐based PSQA were compared to the planned dose distributions using a 2%/2 mm and 3%/1 mm gamma criteria, both with a 10% threshold and a 90% passing criterion. For the SRS plans, 2%/2 mm was used for validation against departmental standards, and 3%/1 mm was used to establish more rigorous QA requirements for SRS type treatments as recommended in recent literature.^36^, ^37^ For an independent dose verification of the TPS, an in‐house Monte Carlo (MC) based second check was used.^38^ To reduce calculation times in our clinical practice, dose calculation grid size is limited to 2 mm × 2 mm × 1 mm (slice thickness) resulting in a 3% statistical uncertainty. Resolution of the MC code only enables use of the 2%/2 mm threshold criteria for comparison; therefore 3%/1 mm values are not reported for the MC second check. Because the training and testing datasets were of unequal size and not normally distributed, nonparametric unpaired methods were used for comparison. The Mann–Whitney U test was used to assess statistical significance, with *p* < 0.05 considered significant. Cliff's delta was used to quantify effect size, and the Kolmogorov–Smirnov test was used to evaluate differences in overall distribution shape and spread. These data are reported as median, interquartile range (IQR), and range. For comparisons between RapidPlan‐generated plans and manual plans, the data were normally or approximately normally distributed; therefore, results are reported as mean ± standard deviation, and a paired Student's *t*‐test was used to assess statistical significance (*p* < 0.05). For comparisons of OAR doses against established dose constraints, conversion to EQD2 resulted in non‐normal distributions for some metrics. In these cases, results are reported as median | IQR (range).

## 3 RESULTS

### 3.1 Dataset comparison

Table 1 shows GTV volume in the test set was slightly higher than in the training set (median 1.00 cc vs. 0.78 cc, IQR 0.74 vs. 0.32), with a statistically insignificant result (Mann–Whitney *p* = 0.061), a small effect size (Cliff's δ = –0.275), and a significant difference in distribution shape (KS *p* = 0.040). The PTV volumes were similar between groups (testing: 2.41 cc, IQR 1.76; training: 2.36 cc, IQR 0.73), with no significant difference (Mann–Whitney *p* = 0.284), negligible effect size (δ = –0.158), and similar distributions (KS *p* = 0.1688). In contrast, the test set showed a significantly larger median distance to the optic nerve (2.40 mm, IQR 0.88) compared to the training set (1.00 mm, IQR 2.70), supported by a Mann–Whitney (*p* = 0.007), a medium effect size (δ = –0.391), and a marked difference in distributions (KS *p* = 0.001).

**TABLE 1:** Training and testing dataset comparison of target structures.

Metric		Testing	Training	Mann– Whitney	Cliff's Delta	Kolmogorov– Smirnov
Number of plans		20	73	‐	‐	‐
Laterality	Left	9	34	‐	‐	‐
	Right	11	39	‐	‐	‐
Location	Anterior	2	2	‐	‐	‐
	Posterior	6	14	‐	‐	‐
	Medial	5	30	‐	‐	‐
	Lateral	5	18	‐	‐	‐
	Superior	2	6	‐	‐	‐
	Inferior	0	4	‐	‐	‐
GTV volume (cc)		1.00 | 0.74 (0.34– 2.46)	0.78 | 0.32 (0.30–2.67)	0.061	−0.275	*D* = 0.340 *p* = 0.040
PTV volume (cc)		2.41 | 1.76 (1.11–5.61)	2.36 | 0.73 (1.26–6.17)	0.284	−0.158	*D* = 0.268 *p* = 0.169
Distance to optic nerve (mm)		2.40 | 0.88 (0.50– 12.00)	1.00 | 2.70 (0.00–15.00)	0.007	−0.391	*D* = 0.468 *p* = 0.001

*Note*: Values reported as median | IQR (range).

### 3.2 Target coverage

Conformality, gradient, and target coverage metrics for manually generated and model‐based ocular SRS plans are summarized in Table 2. No statistically significant differences were observed between planning approaches for RTOG conformity index, Paddick conformity index, gradient index, homogeneity index, global maximum dose, or GTV and PTV coverage metrics, including D99% and mean dose.

**TABLE 2:** Plan quality and target coverage metric comparisons between manually generated ocular SRS plans and RapidPlan generated plans.

Metric	Manual planning	RapidPlan	*p*‐value
RTOG CI	1.08 ± 0.06 (1.00–1.21)	1.07 ± 0.06 (1.00–1.23)	0.723
PCI	0.84 ± 0.05 (0.75–0.90)	0.84 ± 0.05 (0.73–0.91)	0.710
GI	3.29 ± 0.39 (2.69–4.08)	3.45 ± 0.39 (2.82–4.42)	0.106
HI	0.24 ± 0.05 (0.11–0.33)	0.24 ± 0.04 (0.17–0.32)	0.723
*D*_max_ (%)	125.00 ± 4.80 (112.16–131.73)	124.85 ± 2.88 (120.49–129.46)	0.916
GTV D_99%_ (Gy)	25.84 ± 0.43 (24.98–26.84)	26.10 ± 0.49 (25.06–27.39)	0.081
GTV D_mean_ (Gy)	28.19 ± 0.63 (26.65–29.30)	28.45 ± 0.48 (27.78–29.42)	0.240
PTV D_99%_ (Gy)	23.80 ± 0.46 (22.48–24.34)	23.59 ± 0.68 (22.37–24.32)	0.325
PTV D_mean_ (Gy)	27.54 ± 0.47 (26.30–28.47)	27.61 ± 0.35 (27.16–28.33)	0.677

*Note*: Values reported as mean ± Std. Dev (range). Statistically significant *p*‐values are in bold.

### 3.3 OAR doses

Table 3 summarizes various OAR dose metrics. Maximum dose to the ipsilateral optic nerve decreased from 7.18 Gy to 4.56 Gy (*p* < 0.001), and mean dose to the ipsilateral lacrimal gland decreased from 11.61 Gy to 6.47 Gy (*p* < 0.001). Maximum lens dose was also reduced with RapidPlan (9.36 Gy vs. 7.64 Gy, *p* = 0.022). Maximum chiasm dose decreased from 0.89 Gy to 0.46 Gy (*p* = 0.002), and maximum brainstem dose decreased from 0.99 Gy to 0.66 Gy (*p* = 0.019). In contrast, maximum brain dose was higher in model‐based plans (7.20 Gy vs. 6.00 Gy, *p* < 0.001), while skin maximum dose did not differ significantly between optimization techniques (24.26 Gy vs. 24.45 Gy, *p* = 0.574).

**TABLE 3:** OAR dose comparisons between manually generated ocular SRS plans and RapidPlan generated plans.

Organ	Manual planning	RapidPlan	*p*‐value
Ipsilateral Optic Nerve D_max_ (Gy)	7.18 ± 1.26 (4.51–9.88)	4.56 ± 1.74 (1.07–7.23)	**<0.001**
Ipsilateral Lacrimal Gland D_mean_ (Gy)	11.61 ± 7.43 (2.85–25.15)	6.47 ± 6.00 (0.69–17.79)	**<0.001**
Ipsilateral Lens D_max_ (Gy)	9.36 ± 5.66 (3.96–27.28)	7.64 ± 6.17 (1.49–27.62)	**0.022**
Brain D_max_ (Gy)	6.00 ± 1.50 (3.55–8.90)	7.20 ± 1.87 (4.56–11.54)	**<0.001**
Brainstem D_max_ (Gy)	0.99 ± 0.43 (0.47–1.72)	0.66 ± 0.37 (0.19–1.28)	**0.019**
Chiasm D_max_ (Gy)	0.89 ± 0.57 (0.29–2.66)	0.46 ± 0.40 (0.14–1.90)	**0.002**
Skin D_max_ (Gy)	24.26 ± 6.65 (6.91–30.67)	24.45 ± 6.64 (5.29–30.15)	0.574

*Note*: Values reported as mean ± Std. Dev (range). The maximum dose is defined as D_0.03cc_. Statistically significant *p*‐values are in bold.

**TABLE 4:** Time and QA comparisons between manually generated ocular SRS plans and RapidPlan generated plans.

Metric	Manual planning	RapidPlan	*p*‐value
Planning times (min)^a^	∼120	15.01 ± 2.18 (11.47–22.46)	**<0.001**
Total monitor units	11 513 ± 1771 (8165–13 853)	10 189 ± 1772 (7094–13 646)	**0.011**
Modulation factor	4.61 ± 0.71 (3.27–5.54)	4.08 ± 0.71 (2.84–5.46)	**0.011**
Beam on times (min)	8.22 ± 1.27 (5.83–9.90)	7.28 ± 1.27 (5.07–9.75)	**0.011**
EPID‐PD QA (2%/2 mm)	99.15 ± 1.45 (93.40–100.00)	99.85 ± 0.22 (99.50–100.00)	0.275
EPID‐PD QA (3%/1 mm)	93.70 ± 4.01 (82.00–98.30)	97.01 ± 2.30 (92.00–99.30)	0.138
MC 2nd check (2%/2 mm)	97.81 ± 1.48 (94.30–99.60)	98.74 ± 0.95 (95.80–99.70)	**0.008**

*Note*: Values reported as mean ± Std. Dev (range). Statistically significant *p*‐values are in bold.

^a^ Planning times only include the plan optimization and calculation time. Organ and optimization structure contouring, and so forth, is not included. Manual planning time is a conservative lower‐end estimate of the planning time for an experienced HyperArc planner(s).

### 3.4 Planning times and quality assurance results

Average optimization time was reduced from approximately 120 min with manual planning to 15.0 min with RapidPlan (*p* < 0.001). As shown in Table 4, the model‐based plans also demonstrated statistically significant reductions in total monitor units (MUs) (10 189 vs. 11 513, *p* = 0.011), modulation factor (4.08 vs. 4.61, *p* = 0.011), and beam‐on time (7.28 min vs. 8.22 min, *p* = 0.011). Quality assurance results were comparable between planning approaches. No statistically significant differences were observed for EPID portal dosimetry pass rates using either 2%/2 mm or 3%/1 mm criteria (*p* = 0.275 and *p* = 0.138, respectively). A small but statistically significant increase in Monte Carlo second‐check agreement was observed for model‐based plans (98.74% vs. 97.81%, *p* = 0.008).

### 3.5 Demonstration of example RapidPlan case

**FIGURE 1:**
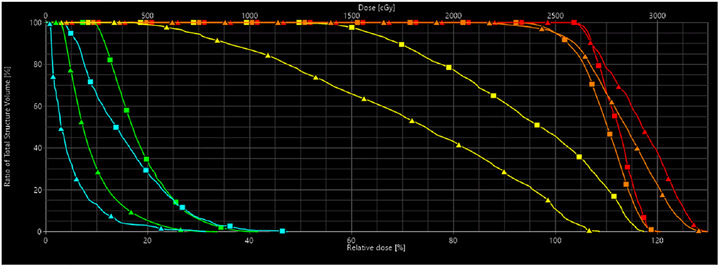
DVH plot, for example right ocular SRS RapidPlan case compared to the manually generated case. Manual plan DVH represented by squares, RapidPlan DVH represented by triangles. (GTV = Red, PTV = Orange, Ipsilateral Lens = Green, Ipsilateral Lacrimal Gland = Yellow, Ipsilateral Optic Nerve = Cyan, Brain = Brown.)

**FIGURE 2:**
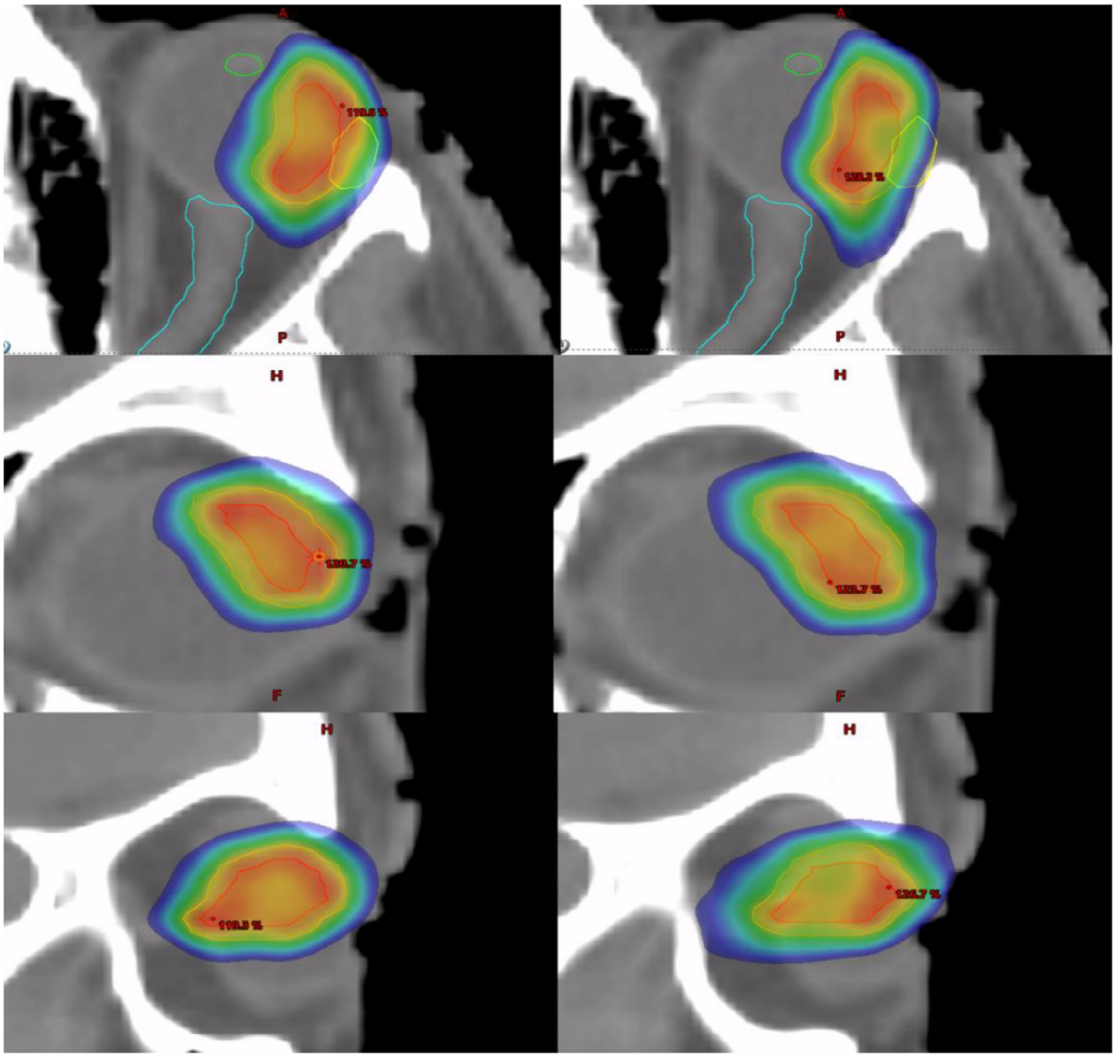
Dose distribution comparison between the manual plan (left) and RapidPlan (right) for left ocular SRS case (as shown in Figure 1). Red upper bound represents global maximum, blue lower bound represents 50% of the prescription. (GTV = red, PTV = orange, ipsilateral lens = green, ipsilateral lacrimal gland = yellow, ipsilateral optic nerve = cyan.)

Figure 1 shows DVH comparisons between a case using a manual planning approach and the model‐based approach. OAR DVHs showed improvement with the model‐based approach. Target DVH curves showed increased dose with RapidPlan as well. Figure 2 qualitatively compares the dose distributions between manual planning and RapidPlan. From Figure 2 we see that RapidPlan preferred a more heterogeneous and less conformal dose distribution to the manual plan to achieve better OAR sparing. Table 5 gives a quantitative comparison between the two planning strategies and is consistent with what we see from Figures 1 and 2. Conformality is decreased and OAR sparing is increased. Target mean dose is increased with RapidPlan. Beam on time and MUs required showed an increase with RapidPlan as well, indicative of more field modulation.

**TABLE 5:** Summary of the example case, reporting plan metrics comparing RapidPlan to a manually generated ocular SRS plan (25 Gy in 1 fraction) via 6MV‐FFF beam.

Metric	Manual planning	RapidPlan
RTOG CI	1.05	1.14
PCI	0.86	0.79
GI	3.29	3.29
GTV mean (Gy)	28.02	29.18
PTV mean (Gy)	27.43	28.33
Total MUs	8519	9676
Beam on time (min)	6.09	6.91
Optic nerve D_max_ (Gy)	8.25	3.96
Lacrimal gland D_mean_ (Gy)	23.50	18.08
Lens (Gy) D_max_	5.67	2.85

### 3.6 Iteratively training RapidPlan model

**FIGURE 3:**
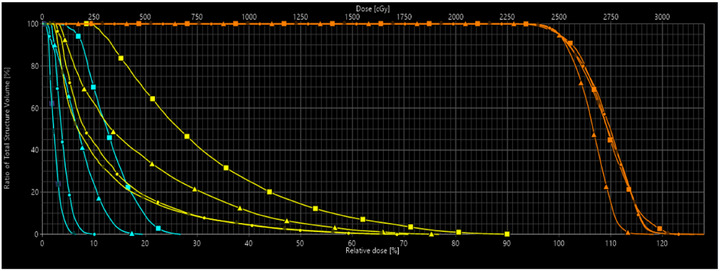
Demonstration of DVH curves for an example testing ocular SRS plan (iteratively) comparing manually generated planning (squares) to various RapidPlan models (V1.0 = triangles, V2.0 = circles, V3.1 (final) = dark circles) (PTV = orange, lacrimal gland = yellow, optic nerve = cyan).

An example test case using a 6MV‐FFF beam is shown in Figure 3 to demonstrate the improvement in model performance over the creation and training of multiple separate RapidPlan models, iteratively. Lower target dose can be seen in the first RapidPlan model (V1.0) but subsequent models achieved similar target dose as the manual plan. Continuous improvements in OAR doses were observed with dose decreasing with the creation of each subsequent RapidPlan model. With each iteration, optimization objectives were removed from almost all contralateral structures as they were not meaningfully contributing to plan performance. The first model contained optimization objectives for 20 structures. The final model has optimization objectives for 14 structures (2 targets, 10 OARs, and 2 control structures).

## 4 DISCUSSION

### 4.1 Dataset comparison

Comparison of the training and testing dataset was done to assess the ability of the RapidPlan model to generalize to other cases. These findings suggest that while GTV and PTV volumes are somewhat consistent between groups, distances to the optic nerve differ meaningfully. GTV volumes differed slightly showing a statistically significant difference in distribution shape from the Kolmogorov–Smirnov test. However, PTV volumes were overall similar between the two datasets as evidenced by no statistically significant difference using Mann–Whitney or Kolmogorov–Smirnov tests. PTV volumes were static expansions of the GTV volumes which allows for more aliasing of the unique GTV shapes and likely explains why GTV targets differed more than PTV targets. The smaller distance to the optic nerve in the training set likely reflects intentional manual creation of targets near the optic nerve, aimed at training the model to handle scenarios where PTVs touch or overlap the optic nerve contour. Training the model for these worst‐case scenarios meant the generalization to cases where targets are further from the optic nerve would be trivial.

### 4.2 Target coverage

Target coverage metrics were comparable between manually generated plans and RapidPlan‐ generated plans, with no statistically significant differences observed in any of the reported metrics. This outcome aligns with the design of RapidPlan, which generates dose‐volume histogram (DVH) estimates and objective constraints for non‐target structures but does not autonomously define target specific dose objectives. Target structure objectives in RapidPlan are manually defined by the model creator and applied uniformly across all plans with the same target doses. In the case of this study, model‐generated plans and manual plans were all normalized to the same level, ensuring that any benefit to OAR doses is largely from model performance, but also that target coverage metrics also did not vary widely between the two methods.

### 4.3 OAR doses

Table 6 shows OAR results from both planning methods compared to established dose limits. Model‐based planning resulted in improved sparing of several critical ocular structures compared with manual planning. Statistically significant dose reductions were observed for the ipsilateral optic nerve, lacrimal gland, lens, brainstem, and optic chiasm, indicating that RapidPlan was able to consistently reduce dose to structures adjacent to the target. It should be noted however, that in the case of the lens, neither planning method was able to consistently meet the OAR constraint. For the optic chiasm and brainstem, the dose differences were statistically significant but occurred at very low absolute dose levels for both planning approaches. In contrast, model‐generated plans demonstrated higher maximum dose to the brain compared with manual plans. Worst case scenarios for the brain maximum dose were still below 12 Gy in both cases indicating either method performs well for sparing the brain. No significant difference in skin dose was observed between planning approaches. Both approaches had plans, specifically those where the PTV included the skin contour, where sparing of the skin may not be possible. Overall, these findings suggest that model‐based planning can improve various OAR doses except for when the target volume includes OAR contours, where in either planning method, target coverage is preferred.

**TABLE 6:** OAR dose constraints and reported doses for manual and RapidPlan‐generated plans.

OAR	Constraint	Endpoint	Manual plan	RapidPlan
Optic nerve^30^	D_max_ < 12.1 Gy	Optic neuropathy	7.18 ± 1.26 (4.51–9.88)	4.56 ± 1.74 (1.07–7.23)
Lacrimal gland^42^	EQD2_3_D_mean_ < 25 Gy	Keratoconjunctivitis sicca	23.85 | 76.42 (3.33–141.59)	4.04 | 38.28 (0.51–73.94)
Lens^42^	EQD2_1_D_max_≤ 10 Gy	Cataract formation	19.10 | 22.00 (6.55–257.16)	19.23 | 28.38 (1.23–263.44)
Brain^43^	V_12_ < 5 cm^3^	Radio necrosis (3.6%)	0^*^	0^a^
Brainstem^42^	EQD2_2_D_max_ < 54 Gy	Permanent cranial neuropathy	0.62 | 0.56 (0.29–1.60)	0.41 | 0.47 (0.10–1.05)
Optic chiasm^42^	D_max_ < 12.1 Gy	Optic neuropathy	0.89 ± 0.57 (0.29–2.66)	0.46 ± 0.40 (0.14–1.90)
Skin^44^	D_max_ < 26 Gy	Grade 3 ulceration	24.26 ± 6.65 (6.91–30.67)	24.45 ± 6.64 (5.29–30.15)

*Note*: D_max_ is defined as D_0.03cc_. Normally distributed data are reported as mean ± standard deviation (range), while non‐normally distributed data are reported as median | IQR (range). EQD2_n_ denotes equivalent dose in 2‐Gy fractions (Gy), assuming an α/β ratio of *n*.

^a^ Worst case maximum dose did not exceed 12 Gy in any plan.

### 4.4 Planning times and quality assurance results

RapidPlan significantly reduced treatment optimization times compared to manual optimizations. Model‐generated plans were within clinical guidelines after a single optimization whereas, manual optimization often required multiple rounds of tweaking optimization objectives to achieve satisfactory plan quality. MUs, modulation factor, and beam‐on times are all related quantities and favored RapidPlan plans compared to manual plans, with statistically significant reductions observed across these metrics (*p* = 0.01). Quality assurance results also showed notable improvements with RapidPlan. All 20 model‐generated plans passed 2%2 mm and 3%/1 mm gamma analysis thresholds. All 20 manual plans passed using the 2%/2 mm threshold, whereas 3 of the 20 manually generated plans failed using the 3%/1 mm threshold. RapidPlan demonstrated a statistically significant improvement in Monte Carlo secondary checks (98.7% vs. 97.8%, *p* = 0.008). These results likely stem from the fact that model‐generated plans were less modulated, increasing TPS and measured dose agreements during EPID based and MC‐based QA.

### 4.5 Comparison to other ocular RP models

Currently, to the best of our knowledge only one instance of a RapidPlan model for ocular treatments has been published in the 5‐fraction SRT setting. Ciernik et al.^39^ compared BrainLabs HybridArc module, which combines dynamic conformal arcs (DCA) with intensity modulated radiotherapy (IMRT), to non‐coplanar VMAT for the treatment of ocular tumors. Patients were treated with 50 Gy in five fractions. The VMAT plans generated by Ciernik et al. used a RapidPlan model trained on 28 training plans and were tested on 13 plans against HybridArc. HybridArc was found to be the superior planning method in their study showing higher conformality and OAR sparing compared to RapidPlan. Their RapidPlan model achieved a median V95% = 95.1% compared to our RapidPlan model V95% = 99.1%. HI was noted as a median of 0.1 compared to our value of 0.24, indicative that the Ciernik et al. plans had more homogeneous target dose distributions as further evidenced by their lower overall global maximum of 107% compared to our 125%. Conformity was improved with our model with a CI = 1.07 compared to Ciernik et al. CI = 1.31. Expressed as a percentage of prescription dose, OAR doses were lower or comparable with our RapidPlan model compared to Ciernik et al. with them reporting optic nerve maximum, lacrimal gland mean, and lens maximums of 62.2%, 25.0%, and 34.0%, respectively. The same values found in our study were 18.2%, 25.9%, and 30.6%, respectively. Dose to optic nerve as a function of prescription dose was improved drastically. It should be noted that direct comparisons between these models are not possible as the results reported are not from the same testing dataset and minor differences in tumor location can drastically change the values reported for model comparison. Additionally, the different fractionations schemes further complicate direct comparisons. RapidPlan model performance was also not a key focus in the Ciernik et al. paper as they were primarily comparing DCA‐IMRT to VMAT. The RapidPlan model used in their paper also was trained on a smaller dataset (28 plans) and no indication was given whether multiple iterations of model training was used. Moreover, their model was not optimized for a specific treatment geometry, as they note that VMAT plans utilized six arcs with 3–5 different couch angles, indicating manual planning was utilized for geometry selection and optimization utilized RapidPlan. In contrast to the model presented here, which was designed and optimized for use with fully automated HyperArc delivery, where selection of treatment geometry is automatic and predictable, and plan optimization is entirely automatic with RapidPlan.

### 4.6 Current limitations and ongoing research

While this study demonstrates a promising ocular SRS HARP model, some limitations should be acknowledged. First, the retrospective nature of this study limits the ability to assess any patient outcome data comparing manual planning to RapidPlan planning. All data sourced for the testing and training datasets were derived from a single institution. Variability was manually added into the training dataset, but this limits the ability to generalize model performance if target characteristics differ greatly between institutions. Additionally, the use of hybrid and synthetic datasets for model training and testing may introduce bias in the recreation of target structures. Special care was taken to make sure the MRI derived tumors were transcribed accurately, and the synthetic tumors were clinically relevant, but we feel this potential bias should be mentioned. Performance of the model and manual planning were also limited by using standard definition MLCs (5 mm width). HDMLCs (2.5 mm width) could further improve both manual planning and RapidPlan performance. Nevertheless, the results obtained with the standard MLC system are promising, suggesting that machines equipped with standard MLCs are still viable for ocular SRS treatments. No fractionation schemes were explored in this study, focusing primarily on a single 25 Gy fraction. However, other fractionation schemes have been utilized for similar treatments like 42 Gy in three fractions or 50 Gy in five fractions SRT. It may be possible to use this single fraction model as a basis for multi‐fraction treatments by removing and prescription specific optimization objectives, Ongoing work is being done at our institution to adapt this model to different fractionation schemes. This study primarily focused on comparing manual planning with a HyperArc‐RapidPlan model results; as such, ocular motion management, a critical concern in ocular SRS/SRT treatments, was not addressed in this study. The use of head‐mounted eye immobilization devices, which are commonly employed,^6^, ^39^, ^40^, ^41^ may not be compatible with the fixed, non‐coplanar, geometry of HyperArc. Our current research is actively addressing these limitations. Clinical implementation of this SRS approach for ocular/intraocular lesions is now underway at our institution, which will facilitate the gathering of a more comprehensive patient data and enable future evaluations of clinical outcomes. Further development of this RapidPlan model is planned for use with HD‐MLCs and risk‐adopted 3–5 fractions SRT treatments based on tumor size, proximity of OAR or magnitude of eye motion as well. Finally, we are planning to develop a motion management strategy tailored for ocular treatments using the HyperArc platform.

## 5 CONCLUSION

A HyperArc‐based RapidPlan model was generated for the treatment of ocular malignancies. The generated RapidPlan model, compared to manual planning, demonstrated similar levels of target coverage and conformality while decreasing dose to adjacent OARs. This iterative RapidPlan model significantly improves plan optimization times of single fraction, 6MV‐FFF plans for the treatment of ocular malignancies using the HyperArc platform. Clinical implementation of this method for ocular SRS is underway at our institution. Currently, work is being done to address motion management concerns via real time eye tracking using a device that is compatible with the non‐coplanar treatment geometry being used, and we intend to share this model so other institutions can further validate its performance.

## AUTHOR CONTRIBUTIONS

DP and CC conceived the research project. CC and SM developed and validated the Ocular SRS KBP model, collected and analyzed the data. DP and WS provided clinical expertise andsupervision of the paper. CC and DP drafted the preliminary manuscript and allco‐authors revised and approved the final manuscript.

## CONFLICT OF INTEREST STATEMENT

The authors declare no conflicts of interest.

## Data Availability

Research data are not shared.
